# A novel technique for flat umbilicus repair in abdominoplasty: bipolar fan-shaped incision and folded suture using paraumbilical flaps

**DOI:** 10.3389/fsurg.2026.1868958

**Published:** 2026-06-25

**Authors:** Xia Linxi, Wang Wenfei, Yang Ruomeng, Wu Chong, Liang Hongwei

**Affiliations:** 1 Department of Burn and Plastic Surgery, Zhengzhou People’s Hospital, Zhengzhou, Henan, China; 2 Department of Burn and Plastic Surgery, Xi’an Central Hospital, Medical School of Xi’an Jiaotong University, Xi’an, Shaanxi, China

**Keywords:** abdominoplasty, bipolar fan-shaped incision, flat umbilicus, folded suture technique, umbilical stalk

## Abstract

**Background:**

The umbilicus plays a pivotal role in abdominal aesthetics, and its reconstruction is essential for achieving a natural and deep appearance that aligns with patients’ aesthetic expectations. However, traditional abdominoplasty has shown limitations in umbilical reconstruction for patients with a flat umbilicus, often resulting in unsatisfactory aesthetic outcomes.

**Objective:**

This study aims to introduce a novel umbilical reconstruction technique, enabling it to create a three-dimensional, stable and aesthetically pleasing navel in patients with a flat umbilicus, so as to improve the overall aesthetic outcomes of abdominoplasty.

**Methods:**

From November 2024 to November 2025, a bipolar fan-shaped incision folded and suture technique was employed in 24 female patients. Specifically, fan-shaped incisions were made at the superior and inferior poles of the umbilical stalk. Following the excision of a moderate amount of tissue, the remaining skin flaps were folded and sutured to construct a three-dimensional umbilical fossa.

**Results:**

This technique is straightforward to perform and has been successfully applied in 24 cases of patients with a flat umbilicus. Postoperatively, the umbilical morphology appeared deep, three-dimensional, and demonstrated stability. Overall, 87.5% (21/24) of patients were highly satisfied with the umbilical appearance, 8.3% (2/24) were satisfied, and only 4.2% (1/24) reported neutral satisfaction due to hypertrophic scars at the superior and inferior poles of the umbilicus. No complications such as umbilical stenosis or skin flap necrosis occurred after the surgery.

**Conclusion:**

The bipolar fan-shaped incision folded and suture technique is an effective method for reconstructing a flat umbilicus during abdominoplasty. In this series of 24 patients, the technique demonstrated a high satisfaction rate and achieved a three-dimensional umbilical appearance with concealed scarring in the short term.

## 1 Introduction

The umbilicus, commonly known as the navel, is the first natural scar inherent to the human body, formed by the healing of the umbilical cord stump after birth. The umbilical morphology holds a distinctive position in public aesthetic perception. For individuals, the umbilicus is not merely an aesthetic unit of the abdominal wall but also a functional unit. The characteristics of an ideal umbilicus encompass its location, size, and shape. Yu et al. (1–3) observed that in young individuals, the umbilicus is typically located approximately 0.7 ± 1.3 cm above the intersection of the abdominal midline and the line connecting the bilateral anterior superior iliac spines, with a length ranging from about 1.5 to 2.5 cm. Furthermore, an aesthetically pleasing umbilicus should feature an umbilical fossa (deep, large, cylindrical in shape, and slightly tilted upwards), an umbilical eave, and be free from visible scarring (4). For abdominal wall surgeons, umbilical reconstruction aims to create a naturally three-dimensional appearance with an appropriately positioned, proportionally sized, aesthetically pleasing umbilicus and concealed scars, thereby achieving an ideal three-dimensional aesthetic outcome (5). Abdominoplasty is a surgical procedure that involves the excision of excess skin and subcutaneous adipose tissue from the abdomen, tightening and suturing of the abdominal wall muscles, along with the reconstruction of a naturally beautiful umbilicus to accomplish the remodeling of abdominal aesthetics.

Craig et al. (6) conducted an analysis of the characteristics of the female umbilicus and categorized its shapes into T-shaped, vertical, oval, horizontal, and distorted forms. Numerous techniques for umbilicoplasty have been described in the literature, including vertical scar flaps, tubular flaps, inverted fan-shaped flaps, C-V flaps, and triangular flaps (7–11). It is found that the most sought-after umbilical morphology is a vertically oriented oval with an umbilical eave (12). However, in clinical practice, a specific subset of patients presenting with a flat umbilicus is frequently encountered. Its typical morphological feature is that the umbilical surface lies flush with the surrounding abdominal skin, appearing flattened without any discernible depression or protrusion. No obvious umbilical fossa can be detected on palpation. This morphological anomaly is predominantly secondary to excessive stretching of the abdominal wall following pregnancy. Traditional reconstruction methods primarily rely on modifying the abdominal flap incisions and approximating the flat umbilicus with a new umbilical flap using a purse-string suture. Such methods often fail to construct a three-dimensional umbilical fossa with natural depth, resulting in an unsatisfactory aesthetic contour and a potential risk of late umbilical flattening. To address these challenges, we have optimized the design of the umbilical stalk and introduced a modified “Bipolar Fan-shaped Incision and Folded Suture Technique” (BFIFS). This surgical approach involves creating fan-shaped incisions at the superior and inferior poles of the umbilical stalk. Following tissue excision, a layered fold and suture technique is employed, which not only enhances the depth of the umbilical fossa but also constructs a biomechanically sound supporting structure. The innovative method provides an effective morphological solution for umbilical reconstruction in patients with flat umbilicus. Clinical applications have demonstrated that the BFIFS has achieved favorable outcomes in 24 cases.

## 2 Patient data

A retrospective review was conducted on 24 female patients who underwent abdominoplasty using the BFIFS technique between November 2024 and November 2025. The case series included patients who underwent abdominoplasty with the BFIFS, completed a follow-up of no less than 6 months, were aged 18–55 years, and had stable body mass index (BMI) with a variation less than 2 for at least three months. Notably, patients with a history of umbilical surgery or incomplete surgical data were excluded. A total of 24 patients were included in this study. Their data, including photographs taken at least 6 months postoperatively, were analyzed.

This study received approval from the Medical Ethics Committee of The Fifth Clinical Medical College of Henan University of Chinese Medicine (Zhengzhou People's Hospital) and was conducted in accordance with the principles of the Declaration of Helsinki. Written consent was provided, by which the patients agreed to the use and analysis of their data.

## 3 Case report

**Figure 1:**
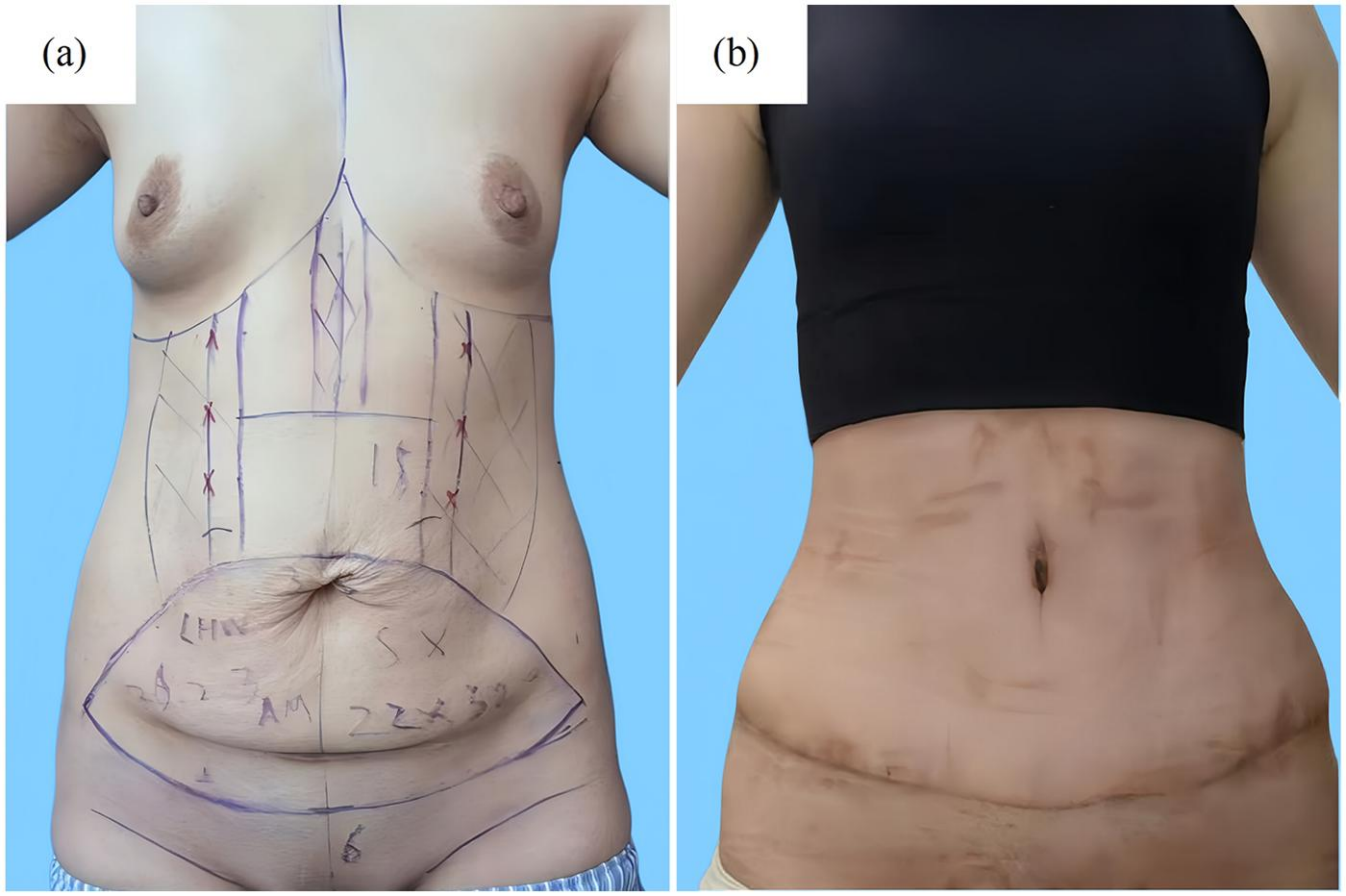
**(a)** Preoperative design and measurement. **(b)** Postoperative outcomes.

A 28-year-old female patient, gravida 3, presented with significant abdominal skin laxity and moderate subcutaneous fat accumulation following childbirth. During abdominoplasty, intraoperative findings showed a complete absence of the umbilical fossa, presenting features of a flat umbilicus (depth: 0 mm). To address this defect, symmetrical fan-shaped incisions were made at the superior and inferior poles of the umbilical stalk. Each incision had a fan angle of about 60°. Subsequently, the skin and subcutaneous tissue were excised along the preoperative markings, followed by layered approximation using 5-0 Vicryl sutures. Finally, the umbilical stalk and the neo-umbilical flap were approximated in layers. The deep layer was closed using 4-0 absorbable sutures, while the superficial layer was closed with 5-0 monofilament sutures. This approach successfully constructed a three-dimensional umbilical fossa structure with a depth of 1.4 cm. Following these procedures, the umbilical morphology exhibited immediate and significant improvement, characterized by the formation of a naturally vertical median umbilical fold. The folded at the superior and inferior poles were symmetrical, and the overall appearance presented a “funnel-shaped” three-dimensional structure that met aesthetic standards. Preoperative design and measurement are presented in Figure 1a, and the 3-month postoperative outcomes are shown in Figure 1b.

## 4 Surgical technique

**Figure 2:**
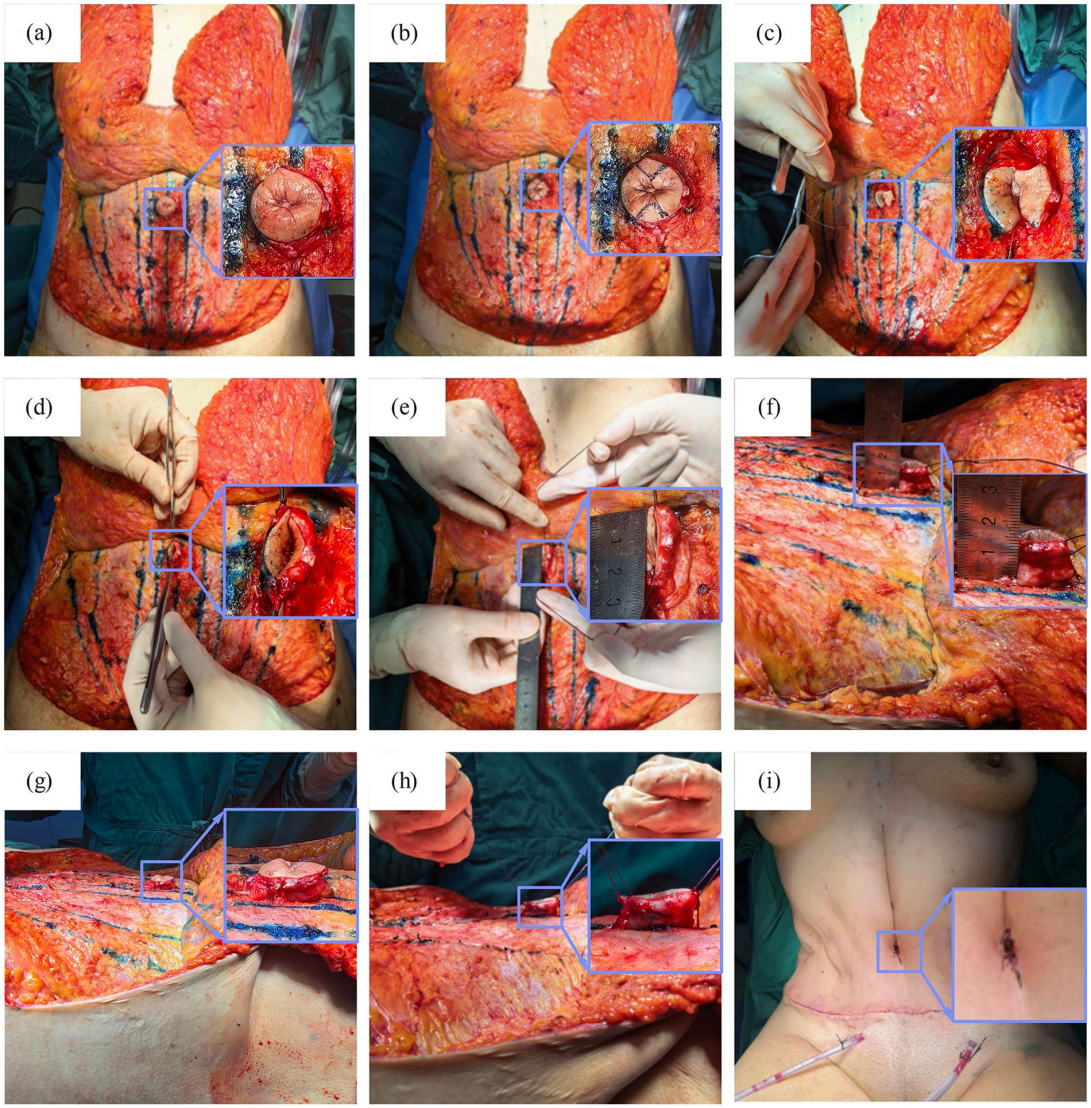
**(a)** frontal view. **(b)** Fan-shaped design. **(c)** Fan-shaped folding. **(d)** Suture finished rendering. **(e)** Umbilical stalk length. **(f)** Umbilical stalk depth. **(g)** Fixation of the superior and inferior Poles of the umbilical stalk. **(h)** Traction test of the umbilical stalk. **(i)** Postoperative frontal view.

After preoperative marking, the patient was brought to the operating room for the procedure under general anesthesia. The patient was placed in a supine position, and the surgical field was prepared and draped. Subsequently, the preserved umbilical stalk was precisely marked and measured. After completing abdominal liposuction and flap elevation, the preserved umbilical stalk was exposed, with bipolar fan-shaped incisions designed at its superior and inferior poles (Figures 2a,b). Skin and partial subcutaneous tissue were excised along the fan-shaped incisions, preserving an adequate volume of subcutaneous tissue. The remaining flap was subsequently folded and closed in layers with 5-0 Vicryl sutures to form a composite flap structure (Figures 2c,d). The vertical length and depth of the umbilical stalk were recorded (Figures 2e,f). The midpoint of the umbilical stalk was suspended to the upper aponeurosis using a 2-0 Vicryl suture. Meanwhile, the superior and inferior poles of the flap were anchored to the linea alba and sutured along the midline of the rectus abdominis aponeurosis. Subsequently, a “traction test” was performed to confirm umbilical elasticity (Figures 2g,h). The patient was then repositioned into a beach chair position (also known as a V-position). Progressive tension sutures were placed along the midline from the xiphoid process towards the umbilicus, terminating just superior to it.

**Figure 3:**
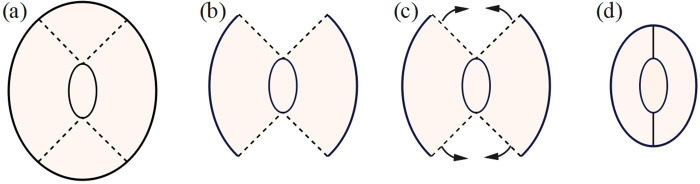
**(a)** Fan-shaped design. **(b)** Resection of redundant periumbilical flaps. **(c)** Fan-shaped folded suture at superior and inferior Poles. **(d)** Final suture appearance.

The new umbilical position was precisely marked on the abdominal flap along the midline using direct visual projection localization, with the incision length of the new umbilicus approximately 2 mm shorter than the vertical dimension of the paraumbilical flap. The skin was incised longitudinally along the marked line using a No. 11 scalpel, followed by thinning of the adipose tissue at the incision margins. Fixation sutures were placed at the 3, 6, 9, and 12 o'clock positions to create a natural concave structure, effectively concealing scars within the umbilical fossa to enhance aesthetic outcomes. Layered closure was then performed: the deep layer was closed using 4-0 absorbable sutures, while the superficial layer was approximated with 5-0 monofilament sutures to ensure precise edge alignment (Figure 2i). The schematic diagram of BIFIFS is shown in Figures 3a-d. Postoperatively, the umbilicus was sterilized, packed with Vaseline gauze, and secured with moderate compression to facilitate morphological remodeling.

## 5 Results

A total of 24 female patients were included in this study. The patients were aged 25 to 44 years, with a mean age of 34.25 years. Their BMI ranged from 17.58 to 29.39, with an average value of 21.90. The mean operative time was 4.3 ± 0.3 h (range: 4.0–4.9 h), with a mean estimated blood loss of 71.8 ± 13.6 mL (range: 50–100 mL). The average length of hospital stay was 8.2 ± 2.1 days (range: 5–12 days). Benefiting from the routine use of patient-controlled analgesia (PCA) pumps for multimodal pain management, postoperative pain was well-controlled. The mean Visual Analog Scale (VAS) score during the initial 48 h was 3.0 ± 1.1 (range: 1–5 points), indicating mild pain. All patients received prophylactic antibiotics (Cefuroxime Sodium) administered 30 min preoperatively and continued for 48 h postoperatively. No cases of wound infection, hematoma, or fat liquefaction were observed. Only one patient developed a postoperative seroma, which resolved successfully after ultrasound-guided aspiration and compression bandaging, leaving no sequelae. The aesthetic outcomes of the umbilicus were evaluated at 6 months postoperatively, with a mean umbilical depth of 1.57 ± 0.18 cm (range: 1.3–1.9 points). Patient satisfaction with the umbilical appearance was assessed using a 5-point Likert scale based on the question, “How satisfied are you with the appearance of your umbilicus?” The evaluation was conducted via patient self-report, with scores assigned as follows: 5 = very satisfied, 4 = satisfied, 3 = neutral, 2 = dissatisfied, and 1 = very dissatisfied. To ensure objectivity and reduce observer bias, the questionnaires were filled out by the patients independently and anonymously, without the presence of the surgical staff. The follow-up rate at 6 months was 100% (24/24 patients), all participants completed the assessment. The results are detailed in Table 1. Regular follow-up visits were scheduled at 48 h, 4 weeks, 3 months, and 6 months postoperatively to observe the healing process and aesthetic results.

**Table 1:** Patient satisfaction with the appearance of the umbilicus after abdominoplasty.

Satisfaction level	Number of patients (*n* = 24)	Percentage (%)
Highly satisfied	21	87.5
Satisfied	2	8.3
Neutral	1	4.2
Dissatisfied	0	0
Very dissatisfied	0	0

The follow-up findings revealed a high overall level of patient satisfaction, with no cases reporting dissatisfaction or extreme dissatisfaction with the surgical outcomes. Specifically, 87.5% (21/24) of the patients expressed high satisfaction with the aesthetic appearance of the umbilicus. 8.3% (2/24) of the patients were satisfied. Only 4.2% (1/24) of the patients rated their satisfaction as neutral due to scar hyperplasia at the superior and inferior poles of the umbilicus. No postoperative complications such as umbilical stenosis or skin flap necrosis were observed. These results confirm that the technique is safe and effective in creating a natural, aesthetically pleasing umbilical shape with a high level of patient satisfaction.

## 6 Discussion

Given the aesthetic significance of the umbilicus, growing emphasis has been placed on its appearance. An ideal umbilicus should feature appropriate positioning and size, a concave shape, and inconspicuous scarring. It is of great necessity to reconstruct an aesthetically pleasing umbilicus through abdominoplasty so as to maximize patient satisfaction.

Current aesthetic trends increasingly favor a small, vertically oval umbilicus. By suturing the umbilical stalk to the rectus fascia and linea alba, we can create adequate depth, draw scars into the depth of the umbilicus, and help maintain a vertical configuration. The umbilical stalk should be trimmed as small as possible (4); with the healing process, the umbilicus undergoes a certain degree of contraction, which not only refines the umbilical appearance but also renders scars more concealed. By trimming and removing the adipose tissue beneath the neoumbilical flap, a vertical incision is made on the neoumbilical flap followed by approximation to the native umbilical stalk, thereby forming a naturally contoured, concave umbilical fossa. Crucially, the abdominal neoumbilical flap must be thinned to a moderate thickness to avoid skin necrosis. Furthermore, the abdominal flap incision should be designed slightly inferior to the preoperative umbilical marking to utilize the suspension effect on the superior stalk upon returning to an upright position, creating a youthful, narrow-superior-wide-inferior umbilical hood. The neoumbilical opening should be approximately 2 mm shorter than the original umbilical stalk to prevent postoperative protrusion or flattening and enhance three-dimensional depth.

The vascular supply to the umbilicus includes the subdermal plexus, perforating branches of the deep inferior epigastric artery, the round ligament, and the median umbilical ligament. Appropriate dissection of the soft tissues surrounding the umbilicus does not compromise its blood supply (13, 14). By suturing the deep dermis of the umbilical stalk to the fascial sheath at the 3, 6, 9, and 12 o'clock positions, the umbilical stalk is further secured at the level of the fascial sheath. While various techniques exist for umbilical reconstruction, our technique was specifically designed to address the lack of depth in flat umbilicus. In our series, this method demonstrated good efficacy in creating a three-dimensional fossa.

In the inclusion and exclusion criteria for this research, patients with a history of previous abdominoplasty or umbilical surgery were excluded. This decision was primarily based on the following considerations: such patients typically have a history of umbilical reconstruction and associated scarring. The resulting potential alteration in local structures would not only significantly increase the complexity of the surgical procedure but could also compromise the viability of the umbilicus due to vascular concerns.

Regarding patients with a history of cesarean section, any disruption to the deep inferior epigastric vascular network caused by the previous lower abdominal incision is considered to have been repaired. Furthermore, the umbilical region possesses a rich vascular plexus, including perforating branches from the rectus abdominis muscle, which ensures stable blood flow to the reconstructed umbilicus (15). The clinical data from this research further corroborates this finding: patients with a history of cesarean section demonstrated outcomes highly consistent with those without such a history. No complications, such as poor wound healing or ischemic necrosis of the umbilicus, occurred. This fully validates the safety and efficacy of this technique in this specific patient population.

Protecting flap perfusion is paramount in abdominoplasty. We perform deep liposuction exclusively in the upper abdomen, combined with selective and precise flap dissection. The resection of the lower abdominal flap is strictly confined to the deep plane of Scarpa's fascia. This approach maximizes blood supply preservation and mitigates the risk of severe complications. A standardized postoperative care protocol was implemented in this study. Regarding compression therapy, patients received to wear medical-grade compression garments continuously (24 h per day) for the first postoperative month. Subsequently, the duration of use was gradually reduced; however, the overall regimen was maintained for at least 6 months to ensure contour stability. In terms of scar management, a comprehensive anti-scarring treatment was strictly adhered to for no less than 6 months. In clinical practice, a first-line combination therapy utilizing silicone gel sheets (Mepiform) and tension-reduction devices was routinely applied. This approach effectively minimized incisional tension and inhibited scar hyperplasia, yielding satisfactory clinical outcomes.

Certainly, several limitations of this study warrant further discussion. First, as a single-center retrospective study, there is an inherent risk of selection bias. The cohort in this study consisted primarily of middle-aged women, a demographic that reflects the current primary clinical demand for abdominoplasty. Currently, no male or elderly patients meeting the inclusion criteria were enrolled. Second, the sample size was relatively small and the follow-up period was short, which precludes a comprehensive assessment of the long-term aesthetic outcomes, such as the risk of late hypertrophic scarring or keloid formation. These factors limit, to a certain extent, the generalizability of our findings to a broader population. Third, the absence of a control group. We did not directly compare the efficacy of the BFIFS technique with traditional umbilical reconstruction methods in patients with a flat umbilicus. Consequently, we cannot definitively attribute the observed aesthetic improvements solely to the specific modifications introduced by the BFIFS, as standard postoperative healing and tissue contraction following any abdominoplasty may also contribute to the final umbilical appearance. In the future, we plan to conduct multicenter, large-sample, prospective controlled studies with extended follow-up periods to further verify the long-term stability and safety of this technique.

Although the BFIFS technique has demonstrated superior aesthetic outcomes in our series, it is essential to objectively contextualize these findings against existing methods. Compared with BFIFS technique, conventional purse-string sutures are rapid but prone to secondary flattening. However, the technical advantages of BFIFS come at the cost of increased complexity. Its primary limitation is the prolonged operative time, as the technique requires precise geometric design and meticulous handling of the dermal-fat flaps to ensure their viability. Nevertheless, for experienced surgeons, the learning curve is relatively short, allowing for rapid mastery and the consistent achievement of excellent surgical results.

## 7 Conclusions

BFIFS technique demonstrates the potential to reconstruct an umbilical structure with adequate depth and stable mechanical support for a flat umbilicus, confirming its feasibility and safety in clinical applications. These preliminary findings suggest that the synergy between physiological anatomical reconstruction and aesthetic optimization is a promising approach. It not only facilitates the maintenance of a three-dimensional umbilical contour but also holds promise for effective scar concealment.

## Data Availability

The original contributions presented in the study are included in the article/Supplementary Material, further inquiries can be directed to the corresponding authors.
